# Patient portal messaging for care coordination: a qualitative study of perspectives of experienced users with chronic conditions

**DOI:** 10.1186/s12875-019-0948-1

**Published:** 2019-05-03

**Authors:** Jennifer L. Hefner, Sarah R. MacEwan, Alison Biltz, Cynthia J. Sieck

**Affiliations:** 1 Department of Family Medicine, College of Medicine, The Ohio State University, Columbus, OH USA; 2 CATALYST, Center for the Advancement of Team Science, Analytics, and Systems Thinking, College of Medicine, The Ohio State University, Columbus, OH USA; 3 Division of Health Services Management and Policy, College of Public Health, The Ohio State University, Cunz Hall, Room 200B, 1841 Neil Ave, Columbus, OH 43210 USA; 4 College of Medicine, The Ohio State University, Columbus, OH USA

**Keywords:** Patient portals, Secure messaging, Patient engagement, Chronic conditions

## Abstract

**Background:**

Patient portal secure messaging (asynchronous electronic communication between physicians and their established patients) allows patients to manage their care through asynchronous, direct communication with their providers. This type of engagement with health information technology could have important benefits for patients with chronic conditions, and a more thorough understanding of the use and barriers of secure messaging among this population is needed. The objective of this study was to explore how experienced portal users engage with secure messaging to manage their chronic conditions.

**Methods:**

Three focus groups were conducted with 17 total patients who self-reported a cardiopulmonary condition. Participants were asked questions about their experience with patient portal secure messaging. Focus group transcripts were coded through inductive and deductive methods to reveal common themes.

**Results:**

Patients’ motivation for using messaging included the speed and ease of such communication and direct access to a physician. Messaging was used by patients as an extension of the office visit and supported coordination of care among providers as well as patient collaboration with family members or caretakers. Patients identified challenges to using messaging, including technological barriers, worry about uncompensated physician time spent responding to messages, and confusion about what constitutes an appropriate ‘non-urgent’ message.

**Conclusions:**

This study highlights the potential of patient portal messaging as a tool for care coordination to enhance chronic disease self-management. However, uncertainty about the appropriate use of portal messaging persists even among experienced users. Additional patient training in the proper use of secure messaging and its benefits for disease self-management may help to resolve these concerns.

**Electronic supplementary material:**

The online version of this article (10.1186/s12875-019-0948-1) contains supplementary material, which is available to authorized users.

## Background

As healthcare delivery has shifted toward patient-centered care, patient portals have become an important health information technology (HIT) that encourages patient engagement by providing access to personal health information. Beyond providing access to a patient’s medical history, patient portals may include a number of other features, including the ability to view test results, make appointments, and send secure messages to providers [1, 2]. In the ambulatory setting, studies have shown that patient portals can help patients better manage their care and improve patient satisfaction [2–5]. Prior research has shown that patients with chronic healthcare conditions have the greatest interest in patient portals [6–8]. This may be due to the potential for HIT in general, and portals specifically, to be an important mechanism to facilitate patient self-management [9–12], which can lead to better control of chronic illness [13–15]. The first large-scale study of the use of patient portals within a health system found that having more chronic conditions predicted both adoption and intensity of patient portal use [16].

Secure messaging is one of the most frequently utilized patient portal features [17]. This feature allows patients to communicate with their providers via electronic messaging (similar to email) about non-emergency questions or concerns and holds significant potential to engage patients in their care. Existing research has found that patients believed that secure messaging could enhance their communication with their providers and that patients were satisfied with secure messaging as a useful way to communicate with their care team [5, 18, 19]. While patients appreciated that secure messaging allowed them to communicate with providers at any time of day [18], they also expressed frustration when providers did not answer their messages in a timely manner [5]. Patients worried that messaging took up too much of their providers’ time and providers were concerned about a lack of clarity in patient messages, messages with inappropriate topics, and patients’ misunderstanding of the proper use of the messaging feature [19].

Studies examining secure messaging specifically are limited. One large-scale study found that the majority of patients used secure messaging and felt it was helpful in managing their care [20], though the few studies of secure messaging’s impact on health outcomes have reported mixed results [21, 22]. In addition, patients managing chronic illnesses who utilized secure messaging wanted additional training in its use and more guidance from providers or healthcare systems about what to expect from secure message communication [19, 20, 23]. These studies offer insight into the experiences of patients engaging in the secure message feature within a portal.

As patients gain more exposure to portals and other HIT, research suggests their experiences and needs change [19, 24]. As such, it is important to study how patient use of this technology evolves with frequent utilization for care management, particularly regarding engagement in asynchronous communication with their primary care physicians. The study presented here addresses this need by examining more deeply the use of secure messaging to manage care among patients with chronic conditions who are engaged in using the portal.

## Methods

### Study design

We conducted an exploratory qualitative study utilizing focus groups to better understand the way patients with chronic conditions utilize secure messaging within the patient portal. Specifically, we conducted three focus groups comprised of a convenience sample of patients with cardiopulmonary conditions (the disease focus of the grant that funded this study).

The semi-structured focus group guide included questions about how patients learned about the portal, how they were initially trained in its use, their frequency of messaging, how they decided when to message, the value of the messaging feature, and thoughts about how messaging impacted their provider. The focus group guide is available as Additional file 1.

### Study setting

This study took place at three of the fifteen Department of Family Medicine clinic sites that are part of a large Midwestern Academic Medical Center (AMC) that uses MyChart (Epic Systems; Verona, WI) – an interactive ambulatory patient portal tethered to the patient’s electronic medical record. MyChart allows patients to access their electronic health record, request appointments and medication refills, and communicate with providers through secure messaging. Secure messaging is a portal feature that enables asynchronous electronic communication (similar to email) between physicians and their established patients. When sending secure messages, patients are shown a notice on the screen telling them to use this feature for non-urgent messages only, to expect a response within 24–48 h, to call 911 if they feel their concern represents an emergency, and to be aware that their message will become part of their medical record.

### Study recruitment

Recruitment took place at three Department of Family Medicine clinic sites within the AMC during their Patient and Family Advisory Council (PFAC) meetings. The PFAC is a voluntary council that meets quarterly at each ambulatory clinic with a goal of engaging clinic stakeholders in discussions about patient satisfaction and clinical processes. PFAC membership consists of current clinic patients and/or caregivers, and clinic stuff such as Medical Assistants, physicians, and the clinic manager.

At each of the three PFAC meetings the principal investigator (JLH) introduced the study and inclusion criteria, distributed informative flyers, and passed around a sign-up sheet for interested patients. Inclusion criteria included current use of MyChart and diagnosis with a cardiopulmonary condition, though this information was self-reported as participation was anonymous. The study protocol was approved by the Institutional Review Board affiliated with the AMC.

### Sample

The inclusion criteria ensured that all participants were established users of MyChart; while it is important to gather the insights of patients who do not use MyChart that is outside the scope of this study. Two of the three study clinics were in suburban locations and one was urban, serving as a safety net site for the AMC. There were 17 participants across the three focus groups; six patient participants were also providers working in Family Medicine clinics (three medical assistants, two nurses, and one practice manager) and therefore also had experience working with MyChart from the provider perspective. The results presented in this study represent the patient perspective unless it is explicitly noted that the comment was from the provider perspective. Recruitment was concluded after the third focus group because of a saturation of themes.

### Focus group process

Each focus group adhered to the characteristics and process outlined by Krueger and Casey [25]. The first group consisted of 5 participants, the second and third had 6. Participants were seated around a comfortable circular table, provided with lunch to eat during the session, and initial introductions were conducted to increase comfortability in the setting. The group was moderated by the principal investigator (JLH), who is experienced in conducting focus groups, assisted by a note taker. Before the moderator began the discussion using the semi-structured focus group guide the participants were taken through a process of verbal informed consent and instructed about group confidentiality. Each participant received a $50 Target gift card as a thank you for donating his/her time.

### Analysis

Focus groups were audio recorded and transcribed for analysis by three medical students employed engaged in summer research projects within Catalyst - our health services research center at the AMC. Transcription was overseen by the principal investigator (JLH) and the master’s trained project manager who developed and supervises student transcription within our center. Following the methods of thematic analysis from Constas [26], an initial codebook was developed by identifying broad themes linked to interview guide questions. The principal investigator (JH), an experienced qualitative researcher who conducted the focus groups, led this coding process in collaboration with the third and last authors (CJS). During preliminary coding using that codebook, a list of emergent codes and subcodes was developed and applied to the transcripts. Each transcript was reviewed by at least two members of the coding team and team members met regularly to clarify any coding discrepancies. We used the qualitative data analysis software program ATLAS.ti (version 6.0) to support coding and exploration of themes within the data.

The study team then engaged a group of five patients in a member-checking process (recruited via email from the pool of 150 existing Patient Advisors at the AMC who currently serve on seven different councils across the medical center and volunteer for other research and quality improvement projects on an as needed basis). During a meeting lasing an hour and a half the principal investigator led the patient advisors through a member-checking process, a method that Guba and Lincoln [27] posit is the most crucial technique for establishing data credibility. Specifically, the patient advisors were provided transcripts of the focus groups and the coding dictionary and they engaged in a coding process and open discussion related to the applicability and validity of the codes in the context of the data collected. This group of patients confirmed understanding and applicability of the coding dictionary.

## Results

### Motivations for using secure messaging to manage conditions

Among patients with chronic health conditions, thematic analysis of focus group transcripts revealed a major theme around the motivations for using secure messaging to manage a chronic condition. Motivations included the fact that secure messaging is quicker than calling the office and provides direct access to a patient’s physician. We present these subthemes below and provide representative quotations in Table 1.

**Table 1:** Patient-reported motivations and uses of secure messaging for disease management

Motivations for using messaging	Representative comment
*Quicker than calling the office*	“I'd rather do that [send a portal message] and wait … I’d rather wait that way as opposed to someone telling me [on the phone] they can’t see me until December.”
	“Well it saves the phone call, the message, the phone call back.”
	“I know that was in the morning, by the noontime I had heard from her via the telephone and she had made arrangements for me to get in to see another doc in this practice so they could do urine specimen but I was able to get some meds quick.”
	“It’s a lot quicker [than the phone].”
*Direct access to a physician*	“Saves phone calls, saves this kind of messages from having to go from who answers the phone to the doctor.”
	“My doctor, she’s really good at checking her email. She says she tries to get in between each person she sees…to see if she has anything new. Which is nice, you know she replies pretty fast.”
	“I didn’t want to call the office to go through the gatekeeper, so I wrote [a portal message] hoping that my doctor would read that today.”
Uses of messaging for care management	Representative comment
*Extension of the office visit*	“It’s mostly kind of just instead of having to come in for an appointment every week.”
	“Like if I’m sending a message like this, it’s not something where I’m like ‘I really need to know this right now.’ It’s like, 'Oh hey I thought about this, this is something I don’t want to have like schedule an appointment, I would just like to know the answer sooner rather than later.'”
*Coordination of care*	“The other thing I love doing it [sending secure messages] for is sometimes I don’t know what doctor to go to, I was seeing so many specialists I really didn’t know. So, I would go to like my doctor since she was managing my care and say, ‘Who do you feel comfortable with me asking, you know going to?’ And she would always just respond right back and say, ‘You should go to your cardiologist for that,’ or pulmonologist or whatever so that was really helpful.”
	“After my initial appointment she said you know, she would send me messages about how she set me up for appointments with the neurologist, cardiac, um and a lung test, um some type of test.”
	“For instance, I’m having a dental procedure done in May and I might have to stop a blood thinner. I’m just going to email my family doctor and say, 'I need your approval that this is okay, you respond to this MyChart message, I'll print it out and I’ll give it to the guy doing the surgery.'”

#### Quicker than calling the office

Patients reported that one of the major reasons they chose to use MyChart’s messaging feature was to avoid having to call their provider’s office. Messaging was generally quicker than calling their provider’s office and potentially speaking to several people about their concern or working their way through a phone tree. For example, one patient noted that she felt like messaging her provider was quicker and less frustrating than having to call: *“I feel like it eases any frustration on all parties like with the you know people answering the phone … I feel like it’s a smoother transition when you’re able to do it through a message than having to call and wait for a call back..”* Another patient described the benefit of asynchronous communication, rather than coordinating a time when both parties are available by phone: *“Oh yeah, playing phone tag, I hate that with some doctors.”*

#### Direct access to physicians

In addition to noting that messaging allowed for quicker contact with a provider, patients also reported that messaging allowed them direct access to their physician. For example, one patient discussed the benefit of being able to talk about her symptoms and get advice directly from her physician: *“It was better [than a call to office staff] because, I was telling her I think I’m having some problems on passing out and you know she was like what kind of symptoms are you having when you’re passing out, and she asked for details … she knows me.”* Another patient summed up the benefit of direct access to physicians via messaging: *“You know it gets to the right person.”*

### Uses of messaging for care management

Interviewees also noted the ways in which secure messaging is used for chronic disease self-management, including as an extension of office visits and to coordinate care between multiple providers. Below these themes are described further with supporting quotes in Table 1.

#### Extension of office visits

Many patients stated that they utilized the MyChart secure messaging feature to avoid having to go in to their physician’s office every time they had a minor problem. One patient explained that this allowed him to avoid frequent follow-ups for his chronic condition: *“So like instead of coming in every time like blood pressure is a little high, we can just message the doctor and see if there’s anything we should be doing or that we think we should change.”* Patients understood the need to keep their primary care physician updated about the status of their chronic condition; sending a secure message was a way to keep their doctor in the loop without scheduling an in-person office visit every time they needed to share information about their condition with their provider.

#### Coordination of care

Several patients also noted that messaging allowed them to coordinate care amongst providers and share information with family members and caregivers. A patient talked about the way she was able to provide her primary physician with information about specialist visits via MyChart secure messaging: *“My doctor would ask me about a response that I had received from another provider and so I could also pass that on to her if she needed that information. So it was really helpful.”* Another patient echoed this idea when discussing the way she utilized secure messaging to determine which provider her husband should see for a procedure:

*“Another thing that was really helpful recently was when you’ve had to have a little procedure done and we could not figure out who was supposed to be doing it or who we contact. So, like I messaged our primary care [provider] and then I messaged our dietician and then I messaged our speech therapist. And like everyone, they kept kind of referring to each other. It was nice because I didn’t have to call anyone, I just kept forwarding the message.”*

One patient discussed the way utilizing messages allowed her to look back at conversations when talking to her husband about her health: *“It helped me if I needed to go back and look at something like I was unclear or my husband wanted to talk about what they said it was a little easier with the online conversation actually cause it was right there in my hand so I could say this is what they said specifically.”*

### Challenges of using securing messaging

Analysis of the data from the patient focus groups also revealed three types of challenges related to using secure messaging for chronic condition management: technical challenges, worry about physician time, and confusion about what constitutes an appropriate ‘non-urgent’ message. Below we present more details on these challenges and provide representative quotes in Table 2.

**Table 2:** Patient-reported challenges of using secure messaging via a patient portal

Challenge	Representative comments
Technical challenges	“Yeah and I’m thinking and I know this population that comes here, the bulk of the population, don’t necessarily use a computer.”
	“I recall when it first came out is that there was a significant percentage of patients who did not have computers … There’s still some that don’t feel comfortable and so some cases they would have a son or a daughter do it for them.”
Worry about physician time	“Because of my background I try to keep it concise and short and non-urgent.”
	“Sometimes I’ll rewrite, maybe I’ll get all wordy and then I’m like that’s too many words. Then I’ll try to be more concise. But then sometimes I’ve found I don’t get necessarily the information I want, sometimes I’m asking for their impression or their feedback about something and maybe that’s not the, I needed to have called for that versus cause it’s not as easy to respond with printed words, I don’t know.”
Determining what constitutes a non-urgent message	“See that’s why I chose not to call the office and take a chance on it being non-urgent, and fortunately my doctor happened to look at hers and responded, but I was prepared just to wait.”
	“He did do a good job of describing what to message about you know anything serious obviously come in.”
	“I had never in my life had anything like that before and I wanted a doctor to say something before I go to emergency.”
	“My idea of MyChart was to communicate with my doctor and everything’s not an emergency like giving her in person my blood pressure but that’s what she requested.”

#### Technical challenges

Several patients reported continued difficulty using the MyChart secure messaging feature. One patient had problems remembering how to send her blood pressure readings to her provider, particularly when she had not used MyChart in a while:

*“I said wow you know I should be sending messages, honestly I forgot how, but I should be sending messages about my blood pressure and I don’t. Like I said, sometimes I’m just technically challenged and I’m just not as comfortable in certain realms now … When I’m not on MyChart as much there’s a level of uncomfortableness. I just don’t send the messages as often as I should.”*

Patients in the focus groups that were also providers echoed this sentiment, noting that low technological literacy and lack of access to computers were some of the major barriers to use of MyChart in their offices: *“But that was the main issue, not all patients were adept or comfortable at all with, or had the means to, use the computer.”*

#### Worry about physician time

Patients also reported concern about the way MyChart messages impacted their physicians, often trying to keep messages short in order to respect their time. A patient explained why she tries to keep initial messages to her provider short: *“Sometimes I’m more vague just cause I know they’re so busy and I figure if they need more information they will always respond back. So I try not to make it like super, super long.”* Patients in the focus group that were also providers acknowledged that long, unfocused messages could be a challenge to work through and could take up large amounts of their time: *“A lot of people when they talk about stuff they go off track and talk about something else so you know that’s going to take up a lot of time for the doctor trying to read and trying to figure out what’s going on.”*

#### Determining what constitutes a non-urgent message

Patients frequently mentioned that they were aware that messaging was not to be used for emergency situations and patients kept this idea in mind when discussing their use of messaging. One patient provided an example of how she uses messaging for non-urgent issues: *“I like being able to communicate that with things that are important … They’re not life threatening.”* Patients in the focus group who were also providers noted that different patients may have different ideas about which issues are appropriate to send messages about and which should be considered too urgent for messaging. A provider emphasized the idea that patients have varying ideas about what constitutes an emergency: *“Right, I mean I guess it’s up to the patient to decide what non-urgent means.”*

## Discussion

This study details the perspectives of patients with chronic conditions on the use of secure messaging via a patient portal for chronic disease management, including motivations for choosing asynchronous electronic communication with a physician, how it is used in care management, and challenges to this type of communication. Patients generally found messaging more convenient and quicker than calling the office with questions and concerns. Additionally, patients found that when managing their chronic condition, messaging could reduce the need for frequent office visits to discuss condition changes and updates. These observations corroborate earlier studies that have found that messaging between patients and providers can result in decreased patient phone calls or office visits [28–30].

A new finding in our study was the use of secure messaging to help manage care between multiple providers. This benefit may be especially useful for patients with chronic conditions, as they often require more frequent communication with multiple providers. Patient portals are already recognized as a valuable HIT tool for patients with chronic conditions [31], and our results suggest secure messaging features may further improve the ways in which patients can manage their healthcare. Use of secure messaging to coordinate care may be particularly relevant as patients interact more frequently with interprofessional teams. Research suggests such teams can improve the quality of communication between patients and providers and positively impact health outcomes [32, 33]. As the use of interprofessional teams continues to grow, more research is needed to fully understand how secure messaging is currently used by patients for care coordination and how patients and providers can be trained to more effectively utilize messaging to coordinate their care between multiple providers and services.

Unlike previous studies [18], patients expressed few concerns about security when using secure messaging. Additionally, most patients reported little difficulty accessing the portal, suggesting that patients who regularly use the portal are already proficient in basic computer and portal use. While both patients and providers have had time to become more comfortable with possible security concerns, we also acknowledge that our focus groups only included patients who were active patient portal users, so their views and opinions may differ significantly from inexperienced portal users or those who have challenges accessing or utilizing this technology. Different resources may therefore be necessary for different user groups, depending on their experience and comfort with using various patient portal features.

Patients with chronic conditions were concerned about misusing their providers’ time and expressed difficulty knowing how long their messages should be and what kind of information should be included, consistent with previous studies [18, 19, 34]. This suggests that while patients had experience using the portal and were comfortable with the process, they continued to struggle with the “rules of engagement” related to appropriate use. Patients were also concerned about what types of problems were appropriate for messaging. Many patients were aware that messaging was not to be used in emergencies, however, there were discrepancies between patients about what constitutes an emergency, suggesting patients need additional information or examples to define what is inappropriate for secure messaging. The persistence of these worries even among those engaged in using the portal users suggests that patients need additional guidance in order to appropriately utilize the messaging feature [19]. Despite the benefits noted here, without such guidance the potential to expand the use of messaging for disease management may not be fully realized.

### Limitations

While all study participants used the portal to manage their own health, several participants also used the patient portal as a member of a healthcare team at the AMC. The opinions of these participants may differ from those of the general population due to their unique perspective and greater experience with patient portals. However, these participants provided other patients in the focus group with perspective into how providers interact with secure messages and shared interesting insights in which they were able to sympathize with both the patient and provider perspectives.

Another limitation is the lack of demographic statistics and portal usage data for focus group participants. Demographic data is not reported to maintain the anonymity of the study participants who are all well-known members of their respective clinic’s PFAC. Portal usage data was not collected because this was a descriptive qualitative study that did not collect data from participants, however, an area of future research is to correlate patient sentiments with usage data to determine if patient sentiments are different at different portal use intensity levels.

## Conclusions

This study highlights the potential of patient portal messaging to enhance chronic disease self-management by increasing patients’ timely access to their primary care physicians, reducing the need for frequent office visits, and supporting care coordination. However, patients also expressed concerns about the appropriate use of portal messaging. Patient training–on paper, in person, or electronically–targeting primary care patients with chronic conditions could provide guidance on appropriate topics for communication through secure messaging and suggest ways to use the messaging feature to enhance disease self-management activities, such as those outlined in this study. Further research is needed to develop this training and study its implementation and efficacy.

## Additional file

Additional file 1: Focus Group Guide. The focus group guide is provided as a supplementary file. (DOCX 25 kb)

## Abbreviations

AMC: Academic Medical Center
HIT: Health Information Technology
PFAC: Patient and Family Advisory Council
