# Supplementary material for: Patient portal messaging for care coordination: a qualitative study of perspectives of experienced users with chronic conditions
Source: BMC Fam Pract. 2019 May 3;20:57. doi: 10.1186/s12875-019-0948-1 (PMC6499960; doi:10.1186/s12875-019-0948-1)
Supplement: Supplementary file 1 — Focus Group Guide. The focus group guide is provided as a supplementary file. (DOCX 25 kb) [file 12875_2019_948_MOESM1_ESM.docx]

**Rules of Engagement**

Patient Focus Group Guide–DRAFT

**OVERVIEW OF INTERVIEW TOPICS**

In this interview, I will ask you a series of open-ended questions to get your perspectives about different topics. As an overview, these topics are:

## Section 1: Information about you.

## Section 2: OSUMyChart use and training.

- Section 3: Concerns about using secure messaging.

## Section 4: Thoughts about potential training

It is very important to respect the confidentiality of everyone in this group. Out of respect to others, please do not share the names of anyone in this group or repeat what we discuss outside of this focus group.

**INTRODUCTION TO THE FOCUS GROUP**

** Consent script inserted here **

# PATIENT FOCUS GROUP QUESTIONS

## Section 1: Patient Information

- To start, could you please tell me a little about you*?*
  - What is your gender?
  - How old are you?
- Do you usually see the same doctor for most of your care?

**Section 2: OSUMyChart** **use and training**

*I’d like you to think about the first time you heard about OSUMyChart.*

- Tell me about how you first learned about OSUMyChart?
  - Who told you about it?
  - What were you told you could do with this tool?
  - How and where did you sign up for an account?
- In the doctor’s office? At home after an appointment?
- When you first used OSUMyChart, do you remember how you felt about it?
  - What features were easy to use?
  - What features were complicated?
- What do you typically use OSUMyChart for now?
  - What features do you use?
  - How long have you been using it?
  - Do you use it to communicate with your doctor
- How do you feel about the messaging feature?
  - Tell me about the messages you send?
- Have you ever used a different portal?
  - At a different doctor’s office?
  - How was it different?

**Section 3: Concerns about using secure messaging**

*When we talk to patients about secure messaging they note a few concerns, and we would like your thoughts on this topic*

- Do you worry about how much time physicians spend on responding to secure messages?
- Do you ever wonder whether providers get paid for the time they spend responding to secure messages?
- Do you ever with you had received more training about what and how to use secure messaging?

# Section 4: Thoughts about potential training

Patients learn to use MC to do things like schedule appointments and look at test results but they also need to know what types of things to include in a secure message and how to decide whether to send a message, call the office or go to the ER. Providers have mentioned the need to train patients on how to use MyChart and the messaging feature.

- How do you feel about the idea of more training?
- What could this training look like?
- When would you want training?
  - When you initially sign up?
  - When you send a message?
- How would you like to get training?
  - In person at the physician’s office? On paper? Online?

# FOCUS GROUP CLOSURE AND FOLLOW-UP

- Is there anything else you would like to tell us about using OSUMyChart and secure messaging?

Again, we would like to remind you that it is very important to respect the confidentiality of everyone in this group. Out of respect to others, please do not share the names of anyone in this group or repeat what we discuss outside of this focus group.

# THANK YOU so much for your time and participation. Your comments were extremely helpful.
